# Arabic version of the Hayling sentence completion test: scale validation, normative data and factors associated with executive functions in a sample of the Lebanese adults

**DOI:** 10.1186/s13005-020-00251-1

**Published:** 2020-12-11

**Authors:** Sarah Boutros, Emilio El Hachem, Joseph Mattar, Souheil Hallit, Hanna Mattar

**Affiliations:** 1 Faculty of Medicine and Medical Sciences, Holy Spirit University of Kaslik (USEK), Jounieh, Lebanon; 2 INSPECT-LB: Institut National de Santé Publique, Épidémiologie Clinique et Toxicologie-Liban, Beirut, Lebanon; 3 Department of Neurology, Notre Dame des Secours University Hospital, Byblos, Lebanon

**Keywords:** Hayling sentence completion test, Executive functions, Inhibitory control, Normative data, Problematic mobile phone use, Air pollution

## Abstract

**Background:**

This study was conducted for several reasons, primarily because of the lack of an Arabic version of the HSCT that could be beneficial in our clinical practice. Another reason is the need to find potential relationships between various factors with executive functions, especially problematic mobile phone use as suggested by many previous studies, since smartphones have become, nowadays, a daily companion of people from all generations. Thus, it is important to conduct this study in Lebanon to be adapted to the ideas, customs and social behavior of the Lebanese citizens. Hence, the objectives of the current study are to use the Arabic version of the HSCT in healthy community-dwelling Arabic-speaking adults in Lebanon, to check its validity compared to other versions of the test, as well as to identify risk factors that might affect the executive functions in these adults.

**Methods:**

Between August–December 2019, 350 participants were randomly selected. The Arabic version of the HSCT, divided into automatic and inhibition conditions, was used; in each condition, participants’ response-time and number of errors committed were recorded.

**Results:**

None of the scale items was removed. For the automatic condition, response-time items converged over one factor (α_Cronbach_ = 0.905) and number of errors converged over seven factors (α_Cronbach_ = 0.334). For the inhibition condition, response-time converged over one factor (α_Cronbach_ = 0.943) and number of errors converged over four factors (α_Cronbach_ = 0.728). Using electricity as a heating method inside the house was significantly associated with a lower response-time, whereas higher problematic mobile phone use was associated with higher response-time. Using wood as a heating system inside the house and higher problematic mobile phone use were associated with higher number of errors, while using Arabian incense (bakhour) inside the house was associated with lower number of errors.

**Conclusion:**

We were able to set normative data for the HSCT Arabic version for use in the Lebanese population. Problematic mobile phone use was associated with lower inhibitory control in terms of response-time and errors number.

## Background

Executive functions (EFs) include the production, preservation, and customization of plans aimed to accomplish the objectives of a given assignment [1]. It includes three main features: (1) inhibitory control, including self-control and interference control, (2) cognitive flexibility, also called mental flexibility, linked to creativity, and (3) working memory [1, 2]. EFs are crucial skills for mental and physical well-being, school and life success, and for cognitive, social, and psychological growth and evolution [2].

Inhibition represents one of the key skills of EFs. It involves the capacity to control one’s attention, behavior, thoughts, and emotions, to suppress a strong internal tendency or external temptation, in order to do what’s more relevant or essential [2]. In the absence of inhibition, people would be controlled by their impulses, settled tendencies of thought or behavior, by the environment that pulls them this way or the other [2]. Consequently, inhibition makes each one of us somehow unique, and allows us to proceed and behave deliberately, instead of being unintentional and impulsive creatures of tradition and routine [2].

Inhibition has become crucial in several research domains within psychology and neurology. It is broadly recognized that inhibitory control is a multidimentional procedure encompassing at least three features, as defined by Bayard S et al.: « (1) Prepotent response inhibition (the ability to deliberately suppress dominant, automatic, or prepotent responses), (2) Resistance to distractor interference (the ability to resist interference from distracting information in the external environment), (3) Resistance to proactive interference (the ability to resist the intrusions of irrelevant information, that were once considered relevant, into our memory). » [3].

Taking into account inhibition as a multidetermined construct emphasizes the exigency to invent validated clinical tools for the evaluation of its features [3]. The Hayling Sentence Completion Test (HSCT), originally developed by Burgess & Shallice, is one of the available tools that evaluate inhibition. It is a measure of prepotent response inhibition that detects frontal lobe dysfunction. In this test, participants hear sentences in which the last word is absent. In the automatic condition, participants are asked to complete sentences correctly (i.e., by a word solidly related to the sentence), reflecting the initiation of a semantically supported automatic response. In the inhibition condition, participants are asked to restrain themselves from saying the cueing word, and to complete the sentence with a totally unrelated word. To perform this task appropriately, participants have to inhibit the related word and its semantic associates [4].

To the best of the author’s knowledge, only five validated languages have been published for the HSCT in adults and elderly people: (1) English [5], (2) Spanish [6], (3) French [3], (4) Italian [7], and (5) Swedish [8]. There are no HSCT normative data for the Arabic speakers to date, so it cannot be used in Arabic-speaking clinical practice and research groups not only in Lebanon but also in all the 25 Arabic speaking countries around the world. Being a very interesting neuropsychological tool, because it is one of the few tests measuring orbitofrontal dysfunction, emphasizes the need to this Arabic version. Therefore, this study intends to create an Arabic version of the HSCT that is standardized to the linguistic and cultural realities of the Lebanese population.

EFs are negatively influenced by many factors, among which cognitive aging, which usually weakens executive functioning [9], and low family socioeconomic status (SES), which predicts a worse performance on tasks that evaluate them [10]. In contrast, high educational levels improve scores in the HSCT [3], and multilingualism is associated with greater amount of controlled attention and inhibitory control, and can play a crucial role in protecting against the deterioration of executive functions with aging by helping to achieve cognitive reserve [11]. Furthermore, there is a positive correlation between healthy diet (e.g. whole grains, fish, fruits and/or vegetables) and executive functions, unlike unhealthy diet (e.g. fast food, sweetened beverages and red meat) that is associated with a decrease in executive functioning [12]. Moreover, a meta-analysis revealed a wide EFs deficit in overweight participants compared to healthy weight controls, specifically a deficit in inhibition and working memory in overweight participants [13], while better physical fitness is shown to improve cognitive flexibility [14]. On another note, a negative correlation was exhibited between EFs and constant exposure to polluted air and traffic noise [15].

Studies concerning the relationship between long-term mobile phone use and EFs are equivocal. Previous findings showed no evidence of a harmful effect of smartphone use on cognitive functioning. Instead, it suggests there could possibly be a favorable effect of phone use on cognitive performance and executive functioning, but the results of longitudinal analyses were confusing [16]. Another study indicated that a daily exposure (2 h/day for 4 weeks) to electromagnetic fields (EMF) emitted by mobile phones (MP) has no effect on executive function [17]. One more research done in 2017 by Hayashi Y et al. reveals that participants who constantly text while driving have low levels of executive function and high levels of impulsivity [18].

Some studies show a relationship between the use of some medications and the increase or decline in executive functions; proton pump inhibitors (PPI’s) have varying degrees of influence on different cognitive domains and have associations with Alzheimer’s Disease (AD) [19]. On another note, Angiotensin I receptor blockers (ARBs) improve memory and executive function in comparison to other antihypertensive drugs, and it attenuates the decline of cognition over time [20]. Moreover, aspirin use is also associated with a preventive effect against cognitive decline, particularly in people at risk for developing dementia [21].

This study was conducted for several reasons, primarily because of the lack of an Arabic version of the HSCT that could be beneficial in our clinical practice. Another reason is the need to find potential relationships between various factors with executive functions, especially problematic mobile phone use as suggested by many previous studies, since smartphones have become, nowadays, a daily companion of people from all generations. Thus, it is important to conduct this study in Lebanon to be adapted to the ideas, customs and social behavior of the Lebanese citizens. Hence, the objectives of the current study are to use the Arabic version of the HSCT in healthy community-dwelling Arabic-speaking adults in Lebanon, to check its validity compared to other versions of the test, as well as to identify risk factors that might affect the executive functions in these adults.

## Methods

### Study design

This study was conducted between August and December 2019. Participants were randomly chosen from the general population across Lebanon by invitations distributed by the municipality of each village from all Lebanese governorates and there were no compensations or rewards to any participant. Individuals included in this study were aged above 18 years old, having no known history of neurological or psychological impairment. Excluded participants were those expected to have abnormal performances on the HSCT, which have been described in a wide variety of neurological, psychiatric, and neurodevelopmental conditions, such as Alzheimer’s disease and mild cognitive impairment [22–24], brain traumatic injury [25], cerebrovascular accidents [26], Parkinson’s disease [27], amyotrophic later sclerosis [28], frontotemporal dementia [28], schizophrenia [29], bipolar disorder [30], and autism spectrum disorder [31]. Participants aged above 55 years old [32] underwent a mini mental state examination (MMSE); those obtaining a score of 24 or more were included in the study [33].

### Minimal sample size calculation

According to Comrey and Lee [34], a minimal sample of 5–10 observations is needed per item of the scale in order to validate a scale. Therefore, a minimum of 300 participants was needed for adequate statistical power, since the assessment of the automatic and inhibition conditions included 15 items each.

### Instruments

Data was collected via a personal interview, and participants were tested with only one examiner in a quiet office. All sections of the questionnaire need approximately 10 min to be completed. The first section assesses the socio-demographic characteristics, including age, gender, region, the number of rooms in the household and the number of persons living in it (both variables used to calculate the household crowding index), the level of education, the number of mastered languages, the monthly income, and the medical history and chronic treatments.

The second section evaluates the diet of each participant, taking into consideration vegetables, fruits, dairy products, breakfast, eating 5 meals per day, sweets consumption, red meat, beverages, and fast food. Each variable is scored between 1 and 5 to get a minimum score of 9 and a maximum score of 45; higher scores indicate a healthier diet. This scale was based on the constituents of the DASH diet (Dietry Approaches to Stop Hypertension) [35].

The third section is the Arabic translation of the short form of International Physical Activity Questionnaire (IPAQ) [36] that asks about three specific types of activities: walking, moderate-intensity activities, and vigorous-intensity activities. Then participants are classified to have low, moderate, or high physical activity.

To be classified as ‘moderate’, participants should match one of the following:

- ≥ 3 days of vigorous-intensity activity for a minimum of 20 min per day.

OR

b) ≥ 5 days of moderate-intensity activity and/or walking for a minimum of 30 min per day.

OR

c) ≥ 5 days of any combination of walking, moderate-intensity or vigorous intensity activities, achieving at least a total physical activity of 600 min/week.

To be classified as ‘high’, participants should match one of the following:

- vigorous-intensity activity ≥ 3 days, achieving at least 1500 min/week.

OR

b) ≥ 7 days of any combination of walking, moderate-intensity or vigorous-intensity activities of at least 3000 min/week.

Participants who did not meet the above criteria were considered to have a ‘low’ physical activity level [36].

The fourth section assesses the exposure to polluted air; participants are asked about the environment where they live, the heating system used (wood, gas, electricity), if they are living or working next to factories (wood, plastic, chemicals …) or power stations, and their exposure to cigarette smoking.

The fifth section represents the Arabic translation of the Short Version of the Problematic Mobile Phone Use Questionnaire (PMPUQ-SV), containing 15 items that are scored from 1 (‘I strongly agree’) to 4 (‘I strongly disagree’), except for 8 items that are reversely scored. Overall scores range from 15 to 60, with higher scores indicating a higher risk for problematic mobile phone use [37].

### The Hayling sentence completion test

The HSCT was translated to Arabic from the French version [3] because it is the only version having sentences likely to be translated to Arabic without big differences or changes and it was adapted to the linguistic and cultural realities of the Lebanese population so the sentences would be clear and potentially have only one answer. It is because of the fact that Lebanon was under the French Mandate until 1943 and thus French became the prevalent non-Arabic spoken language by Lebanese citizens until recently, which means that a large percentage of the population likely speak Arabic and French, resulting in more familiarity with its linguistics characteristics.

The A-HSCT consists of two conditions (automatic and inhibition), for which two different groups of 15 sentences are given. In both conditions, the interviewer reads out loud the unfinished sentences, and the participant has to complete each sentence with one word [4].

#### Automatic condition

The participants are requested to give a word that is related to the beginning of the sentence, and should do so as quickly аs possible. For example, “He mailed the letter without а*.* .. (participant says) stamp.” Time latency in automatic condition measures the participant’s rapidity in initiating an automatic response. According to the scoring system, three error points are scored when participants provide an incorrect word, one point when the answer is semantically related to the sentence, and no error point if the correct word is given. Higher error score corresponds to a lower performance [4].

#### Inhibition condition

The participants are requested to complete the sentence аs fast as they can with a word that is completely unlinked to it, which makes no sense at all in the context of the sentence. For example, “The captain wanted to stay with the sinking. .. (pаrticipant says) apple.” If at any time during this condition the participant completes the sentence correctly instead of using an unrelated word, s/he is told thаt the word is too linked to the sentence and is retold the task instructions. Time latency in inhibition condition gives information about the time needed to inhibit the correct response and find an incorrect one [4]. According to the scoring system, three points are given when the sentence is completed with the answer that fits with it. One point is given when a participant gives an antonym, a semantically related word, or a word that makes a vague reference to the sentence. Participants receive zero points when a totally unrelated word is provided. A higher error score indicates a lower performance [4].

The total time to complete both conditions of the A-HSCT was approximately 5 min. The automatic condition was tested prior to the inhibition section. Two practice sentences were initially presented prior to each condition. Time of response latency was measured and collected using a stopwatch in both conditions; the timing began soon after the tester finished the sentence, and was stopped soon after the participant began their answer. Response latencies were recorded in whole second units and were not rounded up. For instance, a time between 0 and 0.99 was scored as 0. An average response latency score of all the individual’s latencies for each condition was then computed based on all responses, including errors. No time limit was given for responding. Errors were also scored to evaluate the efficacy of the strategy elaborated by the participant to give an incorrect response [4]. The score calculation method for the both conditions is mentioned in the associated appendix.

All sections of the questionnaire were translated to Arabic by a certified translator, then the translation was retranslated to its original language by another specialist. Upon fulfillment of this procedure, the translators compared the versions of every scale to determine whether the variables had the same meaning. No major incompatibilities were found between the two versions for all scales; they were resolved by consensus.

### Statistical analysis

Statistical Package for Social Science (SPSS) version 23 was used for the statistical analyses. Two different methods were used to confirm the HSCT questionnaire construct validity. A principal component analysis, using a promax rotation since the questions of the scales were correlated, was conducted in order to validate the automatic and inhibition parts of the HSCT. Adequacy of the sample was confirmed through the Kaiser–Meyer–Olkin (KMO) index, Bartlett’s Chi-square test of sphericity and scree plot. Factors with an Eigenvalue higher than one were retained. Second, a confirmatory factor analysis was carried out on Sample 2. To assess the structure of the instrument the maximum likelihood method for discrepancy function was used. Several goodness-of-fit indicators were reported: Relative chi square (× 2/df), Root Mean Square Error of Approximation (RMSEA), Goodness of Fit Index (GFI) and the Adjusted Goodness of Fit Index (AGFI). The index of goodness of fit was calculated by the value of × 2 divided by the degrees of freedom (× 2/df) (cut-off values < 2–5). The RMSEA tests the fit of the model to the covariance matrix. As a guideline, values of < 0.05 indicate a close fit and values below 0.11 an acceptable fit. The GFI and AGFI are chi-square-based calculations independent of degrees of freedom. The recommended thresholds for acceptable values are ≥0.90 [38]. The Student t-test was used to compare two means, whereas the Pearson correlation was used to study the association between two continuous variables. Multivariable linear regression models were done to explore factors associated with the response-time and the number of errors taken as dependent variables and taking all variables that showed a *p* < 0.05 in the bivariate analysis as independent variables. A *p* < 0.05 was considered significant. Reliability was assessed using Cronbach’s alpha.

## Results

Out of 364 participants approached, 350 (96.15%) accepted to enroll in this study and completed the HSCT in approximately 5 min. The mean age of the participants was 45.72 years, with 177 (50.6%) of them being females. Other sociodemographic and characteristics of the participants are summarized in Tables 1 and 2. In the automatic condition of the HSCT, 346 participants (98.9%) made ≤2 errors and 333 individuals (96%) responded in ≤2 seconds. Accordingly, no additional calculations were conducted on these scores.

**Table 1:** Sociodemographic characteristics of the participants

Variable	Mean ± SD
Age (in years)	45.72 ± 21.15
Body Mass Index (kg/m^2^)	24.64 ± 3.82
Years of study	16.49 ± 5.79
House crowding index	0.99 ± 0.45
Problematic mobile phone use	31.04 ± 10.84
Total food score	34.65 ± 4.53
	**N (%)**
**Governorate**	
Beirut	42 (12.0%)
Mount Lebanon	66 (18.9%)
North	106 (30.3%)
South	67 (19.1%)
Bekaa	69 (19.7%)
**Classification executive function- time**	
Pathologic	17 (4.9%)
Limit	45 (12.9%)
Normal	221 (63.5%)
Superior	55 (15.8%)
Very superior	10 (2.9%)
**Classification executive function- number of errors**	
Pathologic	16 (4.6%)
Limit	49 (14.0%)
Normal	205 (58.7%)
Superior	72 (20.6%)
Very superior	7 (2.0%)
**Categories of the diet levels**	
Not healthy	2 (0.6%)
Moderately healthy	16 (4.6%)
Healthy	168 (48.0%)
Very healthy	164 (6.9%)

**Table 2:** Number of participants according to the different age ranges

Age range	Number of participants
18–30	132
31–40	26
41–50	40
51–60	67
61–70	32
71–80	30
81–90	21
91–100	2

All the following results will be concerning the second part of the HSCT (The inhibition condition). The results of the inhibiton condition of the HSCT showed that 221 (63.5%) of the participants had normal response-time, whereas 205 (58.7%) had normal scores in terms of number of errors.

The norms for participants classification and the calculation method of the test score are summarized in Table 3.

**Table 3:** Hayling sentence completion test scores calculation [3]

A first transformation of results (response time and number of errors for each participant) via (LOG_10_ + 1) ➔ A for response time and B for number of errors.	
Second transformation using formulas mentioned in the French version: a. *Response time: ŷ* = A + (0.002x age) - (0.008x education) b. *Number of errors: ŷ* = B + (0.003xage) - (0.02x education)	
Z score calculation using the second transformation	
Classification depending on Zscores: a. ≤ −1.65 *pathologic;* b. [−1.64 → -1] *limit*; c. [− 0.99 → + 0.99] *normal*; d. [+ 1 → + 1.64] *superior*; e. ≥ + 1.65 *very superior.*	

### Validation of the HSCT scale

#### Exploratory factor analysis

The principal component analysis (PCA) for the automatic and inhibition response-time and number of errors questions was run over sample 1 (*n* = 250). None of the automatic condition response-time items was removed and converged over one factor (variance explained = 47.92%; KMO = 0.936; Bartlett sphericity *p* < 0.001; α_Cronbach_ = 0.905). Two items (items 3 and 12) of the automatic condition number of errors items were removed since all participants had the same answer. The items converged over seven factors (variance explained = 71.41%; KMO = 0.464; Bartlett sphericity *p* < 0.001; α_Cronbach_ = 0.334). None of the inhibition condition response-time items was removed and converged over one factor (variance explained = 56.59%; KMO = 0.962; Bartlett sphericity *p* < 0.001; α_Cronbach_ = 0.943). One item (item 12) of the inhibitory condition number of errors items was removed since all participants had the same answer. The items converged over four factors (variance explained = 49.57%; KMO = 0.797; Bartlett sphericity *p* < 0.001; α_Cronbach_ = 0.728) (Table 4).

**Table 4:** Principal component analysis results using the promax rotation

Item number	Factor 1	Factor 2	Factor 3	Factor 4	Factor 5	Factor 6	Factor 7
**Scale 1: Automatic condition response time.**							
12	0.818						
7	0.810						
1	0.732						
15	0.731						
11	0.728						
5	0.716						
10	0.716						
4	0.686						
8	0.682						
9	0.676						
14	0.635						
3	0.621						
13	0.614						
2	0.598						
6	0.569						
**Scale 2: Automatic condition number of errors.**							
5	0.685						
7	0.634						
6	0.500						
4		0.572					
15		0.422					
14			0.579				
9			0.502				
13				0.728			
2					0.709		
8					0.709		
1						0.729	
10						0.685	
11							0.816
**Scale 3: Inhibitory condition response time.**							
12	0.823						
6	0.811						
3	0.803						
4	0.779						
13	0.770						
11	0.769						
15	0.762						
5	0.758						
2	0.757						
7	0.750						
14	0.750						
10	0.738						
9	0.731						
8	0.666						
1	0.586						
**Scale 4: Inhibitory condition number of errors.**							
11	0.744						
9	0.713						
10	0.669						
8	0.459						
6		0.791					
1		0.660					
5		0.442					
3			0.746				
2			0.718				
7			0.449				
4			0.425				
14				0.705			
13				0.665			
15				0.528			

#### Confirmatory factor analysis on sample 2

A confirmatory factor analysis was run on sample 2 (*n* = 100), using the structure obtained in Sample 1. The following results were obtained: the Maximum Likelihood Chi-Square = 642.6 and Degrees of Freedom = 270, which gave an × 2/df = 2.38. For non-centrality fit indices, the Steiger-Lind RMSEA was on 0.12 [0.108–0.139]. Moreover, the Joreskog GFI equaled 0.767 and AGFI equaled 0.711.

### Bivariate analysis

The results of the bivariate analysis of factors associated with the response time showed that diabetic patients (− 0.42 vs 0.15), those with hypertension (− 0.42 vs 0.20), those with cardiac diseases (− 0.53 vs 0.08), those taking aspirin (− 0.58 vs 0.11), and those who use electricity as a heating system in the house (− 0.25 vs 0.21) had significantly lower response-time compared to the opposite groups. In terms of number of errors, the results showed that those who do not use wood as a heating system in the house (− 0.18 vs 0.29) and those who use bakhour (Arabian incense) (− 0.25 vs 0.16) had significantly lower number of errors (better performance) compared to the opposite groups (Table 5).

**Table 5:** Bivariate analysis of factors associated with the response time and number of errors

Variable	Response time	Number of errors
**Diabetes Mellitus**		
No	0.15 ± 0.98	0.23 ± 1.02
Yes	−0.42 ± 0.93	−0.06 ± 0.95
*P*	**< 0.001**	0.301
**Hypertension**		
No	0.20 ± 0.97	0.07 ± 1.05
Yes	−0.42 ± 0.94	−0.13 ± 0.88
*P*	**< 0.001**	0.039
**Cardiac diseases**		
No	0.08 ± 0.98	0.02 ± 1.03
Yes	−0.53 ± 1.01	− 0.15 ± 0.74
*P*	**< 0.001**	0.132
**Proton pump inhibitors**		
No	0.06 ± 1.01	0.04 ± 1.04
Yes	−0.27 ± 0.93	−0.20 ± 0.75
*P*	0.024	0.039
**Aspirin**		
No	0.11 ± 0.97	0.05 ± 1.03
Yes	−0.58 ± 0.94	−0.26 ± 0.79
*P*	**< 0.001**	0.015
**Heating inside the house- wood**		
No	−0.004 ± 1.01	−0.18 ± 1.04
Yes	0.007 ± 0.98	0.29 ± 0.86
*P*	0.796	**< 0.001**
**Heating inside the house- Electricity**		
No	0.21 ± 0.94	0.08 ± 0.94
Yes	−0.25 ± 1.01	− 0.09 ± 1.06
*P*	**< 0.001**	0.111
**Bakhour**		
No	−0.04 ± 0.98	0.16 ± 0.95
Yes	0.06 ± 1.03	−0.25 ± 1.03
*P*	0.484	**< 0.001**
**Living in a polluted area**		
No	0.07 ± 0.88	−0.36 ± 1.09
Yes	−0.003 ± 1.03	0.10 ± 0.95
*P*	0.789	0.003
**Exposed to sand and dust soil**		
No	−0.75 ± 0.62	−0.15 ± 1.07
Yes	− 0.16 ± 1.15	−0.05 ± 0.91
*P*	0.028	0.474
**Factories (usines)**		
No	0.003 ± 0.99	0.04 ± 0.99
Yes	−0.05 ± 1.17	−0.66 ± 0.78
*P*	0.692	0.002

Numbers in bold indicate significant *p*-values

Higher age was significantly associated with lower response-time, whereas higher problematic mobile phone use was significantly associated with higher response-time and higher number of errors (worse performance) (Table 6).

**Table 6:** Bivariate analysis of continuous variables associated with the response time and number of errors

Variable	Response time	Number of errors
Age	−0.345^a^	−0.053
Body Mass Index	−0.153^b^	−0.06
Number of years of study	0.145^b^	−0.079
Total food score	−0.052	0.139^b^
Number of smokers in the house	−0.135^b^	−0.077
Problematic mobile phone use score	0.401^a^	0.215^a^
Household crowding index	−0.115^c^	0.012

^a^*p* < 0.001; ^b^*p* < 0.01;^c^*p* < 0.05

### Multivariable analysis

The results of a first linear regression, taking the response-time as the dependent variable, showed that using electricity as a heating method inside the house (B = -0.3) was significantly associated with lower response-time (better performance), whereas a higher problematic mobile phone use (B = 0.03) was significantly associated with higher response-time (worse performance) (Table 7, Model 1).

**Table 7:** Multivariable analysis

Variable	Unstandardized Beta	Standardized Beta	***p***	95% Confidence Interval	
**Model 1: Linear regression taking the response time as the dependent variable.**					
Heating in the house- Electricity (yes vs no*)	−0.30	− 0.15	0.003	− 0.50	−0.10
Problematic mobile phone use	0.03	0.35	< 0.001	0.02	0.04
**Model 2: Linear regression taking the number of errors as the dependent variable.**					
Heating in the house- Wood (yes vs no*)	0.41	0.20	< 0.001	0.20	0.63
Bakhour use in the house (yes vs no*)	−0.29	−0.14	0.007	−0.50	−0.08
Problematic mobile phone use	0.02	0.21	< 0.001	0.01	0.03

^*^Reference group

The results of a second linear regression, taking the number of errors as the dependent variable, showed that using wood as a heating system inside the house (B = 0.41) and higher problematic mobile phone use (B = 0.02) were significantly associated with higher number of errors (worse performance), whereas using Arabian incense (bakhour) inside the house (B = -0.29) was significantly associated with a lower number of errors (better performance) (Table 7, Model 2).

Since sociodemographic variables didn’t remain in the final model, the multivariable analysis results were considered adjusted over them (age, sex,educational level, number of rooms in the household).

## Discussion

To the best of the authors’ knowledge, this is the first study to set normative data for the Hayling test in Arabic, and to assess factors associated with executive functioning among a sample of the Lebanese population. These results constitute the exclusive source of norms for this test in the Lebanese population.

### Validation of the A-HSCT

In this current study, the preliminary results suggest the validity of the Arabic version of the Hayling Sentence Completion Test (A-HSCT) designed precisely for the Lebanese population. Results delivered primary evidence supporting the accuracy and validity of this test as a clinical instrument to measure prepotent response inhibition in Lebanese adults having a wide range of neurological or psychological disorders. In this study, all the sentences were translated to Arabic to be adapted to the Lebanese culture and habits. Consequently, the Lebanese protocol revealed that the new set of sentences of the HSCT preserved strong accuracy, based on Cronbach’s alpha.

As compared to the Spanish version where the Cronbach’s alpha was 0.864 for the response-time in the automatic condition, in the current study, a slightly higher value of 0.905 was obtained. In addition, Cronbach’s alpha for the response time in the inhibition condition in the Spanish version was 0.797, while in this version, it is considerably higher with a value of 0.943.

The Cronbach’s alpha for the number of errors in the inhibition condition of the Spanish version was 0.839. In this Arabic version a slightly smaller value of 0.728 was acquired. In this Arabic version, Cronbach’s alpha for the number of errors in the automatic condition is 0.334 (where items were converged over eight factors). However, the one for the Spanish version in this same condition was not mentioned [6].

### Factors associated with executive functions

#### Problematic mobile phone use

Many studies investigating the effect of Electromagnetic Field (EMF) exposure in mobile phone users, which are waves emitted by mobile phones, report a slower electroencephalograhic (EEG) activity in these individuals, along with a hypoactivation of a major participant in the regulation of executive functions, the Anterior Cingulated Cortex (ACC). Thus, worse performance on tasks requiring executive functions is associated with long-term mobile phone use [16, 18]. In addition, a research studying the effect of EMF on the neurodevelopment of neonates and children highlights the importance of epigenetic mechanisms that can lead to altered attention, memory and cognition [39]. This goes hand in hand with the results of our study, which showed an increase in both response-time and number of errors in the inhibition condition of the Arabic version of the HSCT for individuals with problematic mobile phone use, indicating a poorer performance. On the other hand, some studies contradict these results, suggesting an enhancement in executive functions among mobile phone users. However, most of these studies reported limitations to their results [17, 40], emphasizing on the need to conduct further longitudinal studies to clarify the true effect of mobile phone use on executive functions.

### Heating system and air pollution

The results of our study concerning the effects of outdoor air pollution on executive functions were inconclusive. However, indoor air pollution attributed to the use of wood burning as a heating system is linked to a lower performance of the interviewed participants, particularly in the number of errors committed in the inhibition condition of the A-HSCT. This negative correlation could be due to the exposure of individuals to air pollutants resulting from wood burning chemical reactions, as mentioned in various studies, particularly CO2 inhalation that is anxiogenic and deleterious for executive functions [41, 42]. In contrast, participants who use electricity as a source of heating in their households performed better in terms of response-time in the inhibition condition of our test, which goes in line with the result mentioned above.

On another note, Bakhour is a widespread spiritual practice in the Middle East region, composed of a vast range of chemical compounds and metals. Opposed to the upstated results concerning indoor air pollution, the use of Arabian incense was associated with a decreased number of errors in the inhibition condition committed during the A-HSCT. A hypothesis may arise, concerning the potential attribution to the spirituality inferred by the use of Bakhour among the participants, which is the subject of many studies that elaborate the benefits of spirituality on executive functions, and its capacity in attenuating the severity of depression [43].

### Other factors

In the bivariate analysis, participants who are older, use aspirin, have cardiac diseases, diabetes, or hypertension exhibited better performance in terms of response-time in the inhibition condition of the A-HSCT. These results contradict the literature that underlines the negative correlation between executive functions and aging [9]. However, they could be explained by the fact that many of the elderly participants reported taking metformin, aspirin, atorvastatin, and angiotensin converting enzyme inhibitors (ACEi) or ARBs, which, when taken as a combination, could potentiate the anti-oxidant effect of each drug, enhancing their neuroprotective effects on the hippocampus [44].

### Clinical implications

The A-HSCT is a clinical tool that measures inhibitory control as part of executive functions, reflecting mainly frontal cortex function. We aim that this version of the HSCT would be used, after its clinical validation, in patients with many neurological or psychological disorders to follow them up throughout the years, and to detect amelioration or deterioration of their scores. Moreover, by comparing the patients’ scores, clinicians can detect the efficacy of their treatment and medications.

### Limitations

Our study is based on the questionnaire that was applied on the participants during an interview. Thus, some of these participants might have felt pressured or shy while being interviewed, leading them to give wrong answers or to perform poorly in the given tasks. An information bias is also possible because of potential misunderstanding. The possibility of recall bias might be considered due to the need of the participants to remember activities of their daily lives. The effect of the recall bias could be differential and may lead to the overestimation or underestimation of effects for some factors, hence the need for prospective studies that overthrow the recall bias and can show more significant and precise association between problematic mobile phone use and inhibitory control as part of executive functions. The extent of exposure to different air pollutants was subjectively evaluated by each participant. Unfortunately, there were no possible means to measure the quantity and time of exposure to each air pollutant. In future studies a bigger sample is needed to reinforce the correlation between problematic mobile phone use and the deterioration of executive functions precisely inhibition. Unluckily, there was no test-retest reliability and no comparison with other measures of inhibition or any other test of executive function. Not to forget that, use of a smaller patient group with inhibitory deficit would have been informative, as this would have clarified whether this translation of the Hayling test could be used to identify participants with known inhibitory deficits. It would be better to take these limitations into consideration in the future studies to obtain more precise and reliable results.

## Conclusion

The preliminary results suggest that the Arabic version of the Hayling Sentence Completion Test is now a valid tool that can be used by clinicians to measure response inhibition in adults and elderly patients in the Lebanese population. These results could suggest a potential association between many factors and inhibitory control. The most significant was problematic mobile phone use and the deterioration of executive functions, mainly inhibitory control, in terms of both response-time and number of errors in the A-HSCT. To the best of the author’s knowledge, the present study is the first to create normative data for the A-HSCT in the Lebanese population.

Furthermore, it should be noted that additional studies are needed to prove a possible harmful effect of mobile phone use on frontal cortex, since it became a primarily used feature in our daily life.

## Data Availability

All data generated or analyzed during this study are not publicly available to maintain the privacy of the individuals’ identities. The dataset supporting the conclusions is available upon request to the corresponding author.

## Abbreviations

HSCT: Hayling Sentence Completion Test
EF: Executive functions
SES: Socioeconomic status
EMF: Electromagnetic fields
MP: Mobile phones
PPI: Proton pump inhibitors
AD: Alzheimer’s Disease
ARB: Angiotensin I receptor blockers
DASH: Dietry Approaches to Stop Hypertension
IPAQ: International Physical Activity Questionnaire
PMPUQ-SV: Problematic Mobile Phone Use Questionnaire
SPSS: Statistical Package for Social Science
KMO: Kaiser–Meyer–Olkin
EEG: Electroencephalograhic
ACC: Anterior Cingulated Cortex
ACEi: Angiotensin converting enzyme inhibitors
PCA: Principal Component Analysis
